# Differences in Intercellular Communication During Clinical Relapse and Gadolinium-Enhanced MRI in Patients With Relapsing Remitting Multiple Sclerosis: A Study of the Composition of Extracellular Vesicles in Cerebrospinal Fluid

**DOI:** 10.3389/fncel.2018.00418

**Published:** 2018-11-15

**Authors:** Fabiana Geraci, Paolo Ragonese, Maria Magdalena Barreca, Emanuele Aliotta, Maria Antonietta Mazzola, Sabrina Realmuto, Giulia Vazzoler, Giovanni Savettieri, Gabriella Sconzo, Giuseppe Salemi

**Affiliations:** ^1^Department of Biological, Chemical and Pharmaceutical Sciences and Technologies, University of Palermo, Palermo, Italy; ^2^Euro-Mediterranean Institute of Science and Technology, Palermo, Italy; ^3^Department of Experimental Biomedicine and Clinical Neuroscience, University of Palermo, Palermo, Italy

**Keywords:** multiple sclerosis, extracellular vesicles, cerebrospinal fluid, lymphocytes, surface markers

## Abstract

This study was designed based on the hypothesis that changes in both the levels and surface marker expression of extracellular vesicles (EVs) isolated from the cerebrospinal fluid (CSF) may be associated with the clinical form, disease activity, and severity of multiple sclerosis (MS). The analyzes were performed on subjects affected by MS or other neurological disorders. EVs, which were isolated by ultracentrifugation of CSF samples, were characterized by flow cytometry. A panel of fluorescent antibodies was used to identify the EV origin: CD4, CCR3, CCR5, CD19, and CD200, as well as isolectin IB4. The Mann–Whitney *U*-test and Kruskal–Wallis test were used for statistical analyzes. EVs isolated from the CSF were more abundant in patients with progressive MS and in those with a clinically isolated syndrome than in all the other groups examined. Furthermore, an important change in the number of EVs and in their surface marker expression occurred during active phases of MS [i.e., clinical relapses and the presence of enhancing lesions on magnetic resonance imaging (MRI)]. In particular, the number of CSF-EVs increased in patients affected by MS during clinical relapse; this finding was associated with a decrease in the number of CD19^+^/CD200^+^ (naïve B cells) EVs. These markers are expressed by immature and naïve B lymphocytes, and to the best of our knowledge, this double staining has never been associated with MS, but their reduction has been observed in patients with another type of Th1 cell-mediated autoimmune disease. In contrast, the presence of lesions in the brain and spine on gadolinium-enhanced MRI was associated with an increase in the numbers of CCR3^+^/CCR5^+^ (subset of CD8 memory T cells), CD4^+^/CCR3^+^ (Th2 cells), and CD4^+^/CCR5^+^ (Th1 cells) CSF-EVs. Two points are worth emphasizing: (i) the data obtained in this study confirm that CSF-EVs represent a potentially promising tool to identify biomarkers specific for different phases of MS; and (ii) Considering the role of EVs in intercellular communication, our results provide some insights that improve our understanding of the relationships among some of the cell types that are mainly involved in MS pathogenesis (e.g., lymphocytes, glia, and neurons).

## Introduction

Extracellular vesicles (EVs) are small double lipid membrane vesicles that are released by all cell types. These specialized structures were recently shown to be involved in intercellular communication via both autocrine and paracrine signaling. EVs represent a method for transferring information between cells (e.g., mRNA, miRNA, proteins, and lipids) located in close proximity or in distant sites through biological fluids (Raposo and Stoorvogel, 2013; Turturici et al., 2014; Gangoda et al., 2015; Ratajczak and Ratajczak, 2016). Indeed, EVs have been observed in almost all extracellular fluids [amniotic fluid, breast milk, urine, bronchoalveolar lavage fluid, cerebrospinal fluid (CSF), saliva, semen, and blood] (Witwer et al., 2013).

Two different types of EVs have been identified based on size and the mechanism of formation: exosomes, which are derived from the multivesicular endosomal cell compartment, and membrane-derived vesicles, which are formed by direct budding from the plasma membrane of the cell (Heijnen et al., 1999; Ratajczak et al., 2006; Candela et al., 2010; Morel et al., 2011; Théry, 2011; Abels and Breakefield, 2016). EVs contain a variety of cell surface receptors, signaling proteins, lipids, and nucleic acid, depending on the donor cell type. Therefore, the functions of EVs rely on the type of the parental cells. However, a selective enrichment of specific molecules has been observed, allowing EVs to have different properties and roles from their parental cells (Del Conde et al., 2005; Li et al., 2013). Several types of interactions between EVs and target cells have been identified. Indeed, the interplay may be mediated by ligand/receptor binding, or may be direct, followed by fusion or endocytosis (Turturici et al., 2014).

Several studies identified EVs in the CSF and described their roles in both physiological and pathological conditions, such as stroke, inflammatory neurodegenerative diseases and multiple sclerosis (MS) (Cherian et al., 2003; Horstman et al., 2007; Verderio et al., 2012). EVs have also been detected in blood samples from patients with MS, suggesting that they represent potential biomarkers that would enable clinicians to monitor disease onset and progression (Verderio et al., 2012; Sáenz-Cuesta et al., 2014a,b).

Multiple sclerosis is an autoimmune disorder of the central nervous system (CNS) that is associated with demyelination and neurodegeneration (Trapp and Nave, 2008). The clinical course of MS is highly heterogeneous, and according to traditional classifications, it ranges from a primary progressive (PPMS) form to the relapsing remitting (RRMS) form that is the most common clinical course (Compston and Coles, 2008) and has potentially different responses to available treatments. Another recently proposed classification subdivides MS courses into progressive (pMS) and relapsing (rMS), based on clinical and instrumental findings, with the understanding that the MS course is a dynamic process and that the subtype of classification may change over time (Lublin et al., 2014). The complex nature of MS poses challenges in diagnosis and monitoring disease activity, progression, and treatment responses. Therefore, extensive research has focused on identifying and validating molecular biomarkers that reflect its heterogeneous clinical course and determine the best treatment option for patients (Sáenz-Cuesta et al., 2014a).

Various studies conducted on the EVs isolated from both blood and CSF of subjects affected by MS have revealed an important role for these organelles in the pathogenesis of the disease (Barreca et al., 2017). EVs released by brain endothelial cells, leukocytes, or myeloid cells are involved in MS pathogenesis, inflammatory progression and lesion repair (Sáenz-Cuesta et al., 2014a). T cell activation might be the first step in the autoimmune reaction in the CNS induced by transendothelial leukocyte migration. As shown in the study by Minagar and colleagues, the concentration of endothelial cell-derived EVs depends on disease progression. In particular, these researchers showed that high levels of endothelial CD31-positive EVs were present in the plasma from patients with MS during disease exacerbation, whereas a reduction in the basal level of these EVs was observed during remission (Minagar et al., 2001). EVs released by platelets have also been detected in patients with RRMS (Sheremata et al., 2008; Marcos-Ramiro et al., 2014).

Verderio and colleagues also reported the involvement of EVs in the inflammatory process. A higher myeloid IB4^+^ EV level was detected in the CSF from patients with RRMS than in healthy controls, and the EV concentration was correlated with gadolinium-positive lesions, a sign of active disease (Verderio et al., 2012).

However, the data on the involvement of EVs in the pathophysiology of MS only concern certain steps of the immunological response, and numerous aspects remain to be clarified. Thus, we planned an exploratory study to assess the expression of specific immunomarkers in EVs in the CSF from patients with MS. This project is expected to identify circulating vesicles in the CSF of patients with MS that can be used as a marker of the disease or of its active status. Therefore, the three major objectives of the study were to compare (1) the concentrations of EVs in the CSF from patients with MS who were diagnosed according to 2010 McDonald criteria (Polman et al., 2011) with subjects with other neurological disorders (ONDs); (2) the percentage of EVs in the CSF from patients with MS carrying different antigens due to their presumptive release by microglia, neurons, and different subtypes of lymphocytes, the main cells involved in MS pathogenesis, with subjects affected by OND; and (3) the concentration and percentage of CSF-EVs positive for specific surface markers (Table 1) isolated from patients with rMS using different parameters (i.e., clinical relapse, lesions on gadolinium-enhanced magnetic resonance imaging (MRI), disease duration, and steroid intake).

**Table 1:** Surface markers used to identify different cell types in patients with MS.

Marker	Cell type	Reference
IB4	Myeloid cells	Verderio et al., 2012
CD4	Th cells	Chitnis, 2007
CD4/CCR5	Th1	Bonecchi et al., 1998; Balashov et al., 1999; Zang et al., 2000
CD4/CCR3	Th2	Uzawa et al., 2010
CCR3/CCR5	A subset of CD8 memory T cells	Ferguson and Engelhard, 2010
CD19	B cells	Hauser, 2015
CD200	Neurons, oligodendrocytes, and a subset of astrocytes	Koning et al., 2007; Koning et al., 2009; Valente et al., 2017
CD19/CD200	Naïve B cells	Rasmussen et al., 2015


## Materials and Methods

### Human Subjects

We included individuals affected by MS or OND that had a planned lumbar puncture for their diagnostic work-up during the period from February 2012 to September 2015. None of the patients had received any disease-modifying drug treatment 1 month prior to the lumbar puncture. For the purpose of the study, we collected 15–20 ml of CSF according to the international guidelines (Teunissen et al., 2009). Subjects were diagnosed with MS or clinically isolated demyelinating syndrome (CIS) according to the 2010 McDonald criteria (Polman et al., 2011). Following the publication of the 2013 Lublin criteria, the clinical course at the moment of the lumbar puncture was reclassified according to these new criteria as rMS and pMS (Lublin et al., 2014).

This research project was approved by the Ethics Committee of Palermo University, and all subjects provided written informed consent.

### Magnetic Resonance Imaging Protocol

In the 15 days before the lumbar puncture, all patients with MS and CIS underwent MRI of the brain with a 1.5 T scanner Signa HDxt (GE Healthcare Medical System, Little Chalfont, United Kingdom) using standardized procedures. The maximum lag-time of 15 days between MRI and lumbar puncture was planned to minimize the risk that the data obtained from MRI and lumbar puncture represented two different phases of the disease, because the mean lifetime of a gadolinium-enhanced lesion is approximately 30 days (Guttmann et al., 1995; Ciccarelli et al., 1999). The neuroimaging protocol consisted of the following conventional sequences: FLAIR, T2, T1, and 2D contrast-enhanced T1-weighted SE/FSE. Cervical and dorsal spinal cord MRI scans were performed with the same 1.5 T scanner and the following conventional sequences: STIR, T2, T1, and 2D contrast-enhanced T1-weighted SE/FSE. Subjects with CIS were followed annually with visits and MRIs to evaluate their conversion to MS until November 2017. Controls affected by ONDs followed the diagnostic path planned according to their suspected illness.

### Sample Preparation

Samples of 15–20 ml of CSF were collected in polypropylene tubes. Each sample was centrifuged at 432 × *g* for 15 min at room temperature. The supernatant was centrifuged at 1,730 × *g* for 15 min at room temperature and then frozen at -80°C until use.

### Isolation of Extracellular Vesicles

The final supernatant was diluted in PBS to a final volume of 40 ml, and EVs were purified by ultracentrifugation at 105,000 × *g* for 90 min at 4°C. The pellet was resuspended in 10 ml of PBS and the sample was immediately frozen and stored at -80°C until use.

### Flow Cytometry Analysis

The number of obtained EVs was determined by flow cytometry with a FACSCanto instrument (BD Biosciences, Erembodegem, Belgium). One microliter of EVs was diluted in a fixed volume of 200 μl of filtered PBS (0.1 μm filter); all samples were analyzed by FACS for 30 s at medium flow rate. Gating was performed as described by Sáenz-Cuesta et al. (2014a). The event number corresponds to the number of EVs present in a specific volume of sample.

Samples were immunostained with a panel of fluorescent dye-conjugated antibodies, and the presence and concentration of specific markers were detected by flow cytometry to identify the cells that released EVs. In particular, we used the following markers in the FACS analysis: FITC-conjugated anti-CD19, PeCy-7-conjugated anti-CD200, Alexa Fluor 488-conjugated anti-CD4, APC-conjugated anti-CCR3, PerCpCY-5-conjugated anti-CCR5, and FITC-conjugated isolectin IB4. Please see Table 1 for the cell types identified by these surface markers.

One microliter of EVs was diluted in a fixed volume (200 μl) of filtered FACS Buffer (2% fetal bovine serum in PBS) and incubated with 1 μl of the different antibodies for 45 min at 4°C in the dark. EVs were then washed by ultracentrifugation, resuspended in 200 μl of filtered PBS and analyzed by flow cytometry. The staining of the selected CDs/CCRs was analyzed in two different experimental sets, according to the fluorochromes used: (1) staining for CCR3, CCR5, and CD4, and (2) staining for CD19 and CD200. For IB4 staining, 1 μl of EVs was diluted in a fixed volume (200 μl) of filtered FACS Buffer (10% fetal bovine serum in PBS with Ca^2+^) and incubated with 2 μl of IB4 for 30 min at 4°C in the dark. EVs were then washed twice, resuspended in 200 μl of filtered PBS with Ca^2+^ and analyzed by flow cytometry.

Unstained controls were used to set photomultiplier tube voltages. Compensation was assessed using OneComp eBeads (eBioscience, San Diego, CA, United States), conjugated with the same labeled IgG isotype as the primary antibodies. IgG isotypes were also used as a negative control to determine the fluorescence background. All samples were analyzed by FACS for 30 s at medium flow rate.

All antibodies were purchased from eBioscience, and isolectin IB4 was purchased from Life Technologies (Thermo Fisher Scientific, Waltham, MA, United States). The results obtained were analyzed with FlowJo V10.1 software (FlowJo LLC).

### Statistical Analysis

Means or medians and the corresponding ranges were used to describe the distributions of all variables. Non-parametric tests were applied for statistical analyzes; in particular, the Mann–Whitney *U*-test was used to compare two subgroups of patients, and the Kruskal–Wallis test was used to compare three or more subgroups of patients. SPSS software (SPSS, Inc., Chicago, IL, United States) was used for statistical analyzes. The two-tailed alpha level was set to *p* < 0.05 to indicate a significant difference. The data were processed using GraphPad Prism software version 7.04 (GraphPad Software, La Jolla, CA, United States).

## Results

### Demographic and Clinical Characteristics of Patients

We obtained EVs from the CSF of 39 subjects with MS, 2 subjects with CIS, and 18 subjects with OND. The 39 subjects with MS were classified according to the 2013 Lublin criteria as follows (Lublin et al., 2014): 35 with rMS and 4 with pMS. The status of each subject with rMS at the moment of lumbar puncture was included in Supplementary Table S1.

Within the rMS subgroup, 19 patients had experienced at least two clinical attacks, 14 reported one clinical attack but, according to the 2010 McDonald criteria (Polman et al., 2011), were diagnosed with MS, 1 reported one clinical attack and, according to the 2010 McDonald criteria (Polman et al., 2011), was not yet considered to be affected by MS at the moment of enrolment. However, after 1 year of follow up, this patient was diagnosed with MS, and according to the new 2017 McDonald’s criteria (Thompson et al., 2017), he was considered affected by MS since the beginning of symptom onset. The last patient was affected by a secondary progressive MS according to the traditional classification (Compston and Coles, 2008) but presented a relapse 16 days before the lumbar puncture. The 19 subjects affected by ONDs were split into two groups: 2 subjects with other inflammatory neurological disorders (OINDs), and 16 subjects with other non-inflammatory neurological disorders (ONINDs). Table 2 summarizes several demographic and clinical features of our sample.

**Table 2:** Clinical and demographic features of the studied sample.

	Patients		Controls		
		
	rMS	pMS	CIS	OIND°°	ONIND°°°
No. of patients	35	4	2	2	16
Sex (female)	25	0	1	0	10
Age°	34; 17–62	38; 27–44	35; 29–41	59; 52–66	53; 24–84
EDSS°	2.0; 0.0–5.5	2.0; 0.0–6.0	1.5; 1.0–2.0	NA	NA
Disease duration (months)°	10; 1–360	17.5; 12–114	1; 1–1	NR	NR
No. of subjects in relapse	23	0	1	NA	NA
No. of subjects taking steroids	19	0	1	0	0
No. of subjects with GAD^+^ lesions on MRI	20	0	0	NA	NA


*rMS, relapsing multiple sclerosis; pMS, progressive multiple sclerosis; CIS, clinically isolated syndrome; OIND, other inflammatory neurological disorder; ONIND, other non-inflammatory neurological disorder; EDSS, expanded disability status scale; GAD, gadolinium; MRI, magnetic resonance imaging; NR, not reported; NA, not applicable. °The values are presented as medians; minima-maxima. °°The two subjects with OIND were affected by myelitis. °°°Four of the subjects with ONIND were diagnosed with a chronic cerebrovascular disease, four with normal pressure hydrocephalus, and one each with acute ischaemic stroke, benign intracranial hypertension, spastic paraparesis, parkinsonism, diabetic polyneuropathy, brain tumor outcomes, unspecified consciousness disorder, and conversion disorder.*

The yield of EVs at the end of the isolation procedure did not permit us to perform all of the planned characterizations of EVs on the entire sample. Consequently, for each type of analysis, a table with the characteristics of each individual study population will be shown.

### CSF-EV Characterization

Once isolated, CSF-EVs were characterized by FACS analysis and Nanoparticle Tracking Analysis (NTA) to confirm their identity. Flow cytometry assays showed that CSF-EVs vary in size but are ≤1 μm in diameter (Supplementary Figure S1A). We employed multi-parameter NTA to further define human CSF-EVs. First, we noted the presence of round vesicles displaying typical Brownian motion (Supplementary Figure S1C and Supplementary Movie S1^1^). Next, we observed that the majority of EVs analyzed on the NanoSight NS300 instrument under light scatter mode showed a size distribution ranging from 105 to 405 nm (mean size 136.3 nm, mode 106.1 nm) (Supplementary Figure S1B). The size of CSF-EVs isolated was similar as observed by transmission electron microscopy (Supplementary Figure S1D). To confirm that we have really isolated EVs, FACS analysis by using anti-Hsp70-FITC conjugated antibodies were carried out demonstrating its presence (Supplementary Figure S1E). Indeed, Hsp70 was considered for many years an exosome marker (Lancaster and Febbraio, 2005; Zhan et al., 2009) and more recently it has been demonstrated that this protein is also present within membrane vesicles (Barreca et al., 2017). In addition, since it has previously demonstrated that the acetylcholinesterase enzyme is enriched in EVs (Rieu et al., 2000; Candela et al., 2010), an acetylcholinesterase activity assay was performed to identify the presence of EVs in our samples (Supplementary Figure S1F). Furthermore, the flow cytometry analysis with Annexin-V, which binds externalized phosphatidylserine (PS), revealed that only a very small percentage of CSF-EVs displayed exposed PS on their surface (Supplementary Figure S1G). The specificity of the assay was confirmed by the result obtained in the absence of calcium. Indeed, under these conditions, the percentage of Annexin-V^+^ CSF-EVs was similar to CSF-EV autofluorescence. To the best of our knowledge, this study is the first to show that the majority of EVs isolated from CSF do not exhibit PS. In fact, almost all EVs contain PS (György et al., 2011), with some exceptions (Connor et al., 2010). Finally, the FACS analysis with pyronin Y, a dye that specifically labels RNA, showed the presence of this nucleic acid within the isolated CSF-EVs (Supplementary Figure S1H).

Although, due to the low amount of isolated material we could not follow all the ISEV suggestions to define EVs (i.e., western blot assays) (Lötvall et al., 2014), NTA, electron microscopy, Hsp70 and AChE presence, the results obtained after Triton X-100 treatment, PS exposure on the outer leaflet in a few samples, all together confirmed EV isolation.

### Increased Concentration of CSF-EV in Patients With rMS in the Course of Clinical Relapse Compared to Patients With Stable MS

**FIGURE 1:**
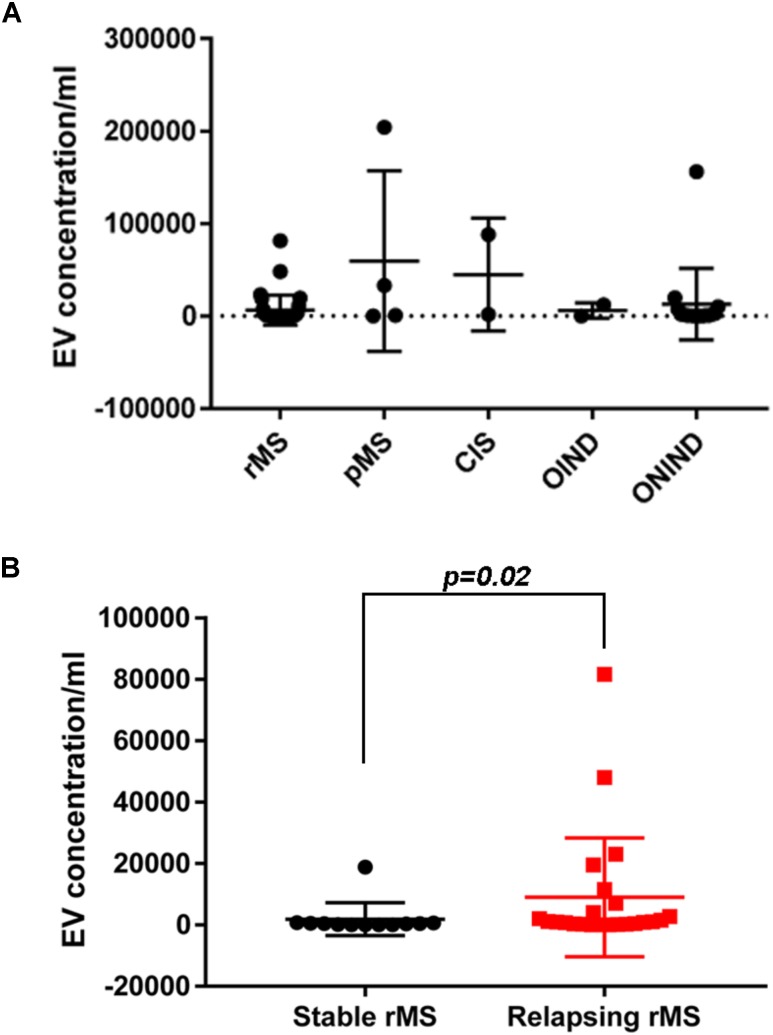
EV concentration in CSF samples from patients with MS and control subjects. **(A)** Quantitative flow cytometry analysis of EVs in human CSF collected from patients with rMS (*n* = 35), patients with pMS (*n* = 4), patients with CIS (*n* = 2), patients with OIND (*n* = 2), and patients with ONIND (*n* = 16). The Kruskal–Wallis statistical test was used to calculate the reported *p*-values (*p* = 0.49). Individual dots indicate values for single donors. **(B)** Quantitative flow cytometry analysis of CSF-EVs collected from patients with rMS in the clinically stable (*n* = 12) or acute (*n* = 23) phase of the disease. The Mann–Whitney *U*-test was used to calculate the reported *p*-values (*p* = 0.02). Individual dots indicate the values for single donors.

We assessed EV concentrations (EV/ml) in whole CSF samples from 59 subjects to determine whether patients with MS and patients with OND had different levels of EVs. Figure 1A shows the mean concentration/ml ± SD of CSF-EV in all examined groups. The analysis of variance and Kruskal–Wallis test did not show any significant difference among the groups (*p* = 0.49).

Subsequently, we evaluated the concentration of CSF-EVs in the rMS subgroup, according to different clinical and instrumental parameters (clinical relapse, lesions on gadolinium-enhanced MRI, disease duration <9 months versus >9 months, and steroid treatment). Figure 1B shows the EV concentration in the CSF samples obtained from subjects during a clinical relapse and subjects who were in a phase of clinical stability (stable rMS). Subjects in the course of clinical relapse showed a significantly higher EV concentration than subjects in the clinically stable phase of the disease. The Mann–Whitney *U*-test showed a significant difference between groups, with *p* = 0.02.

We did not observe significant differences among the other subgroups analyzed (data not shown).

### Equal Percentage of EVs Positive for IB4 in Patients With MS and Patients With Other Non-inflammatory Neurological Diseases

We proceeded from the hypothesis that EVs present in the CSF of patients diagnosed with different clinical forms of MS and OND displayed dissimilar biomarkers on their surface. This information might be relevant to monitor the progression of the disease and to discriminate between MS and OND. The evaluation of this hypothesis was conducted by monitoring biomarker levels present in circulating CSF-EVs from patients with different diseases.

**FIGURE 2:**
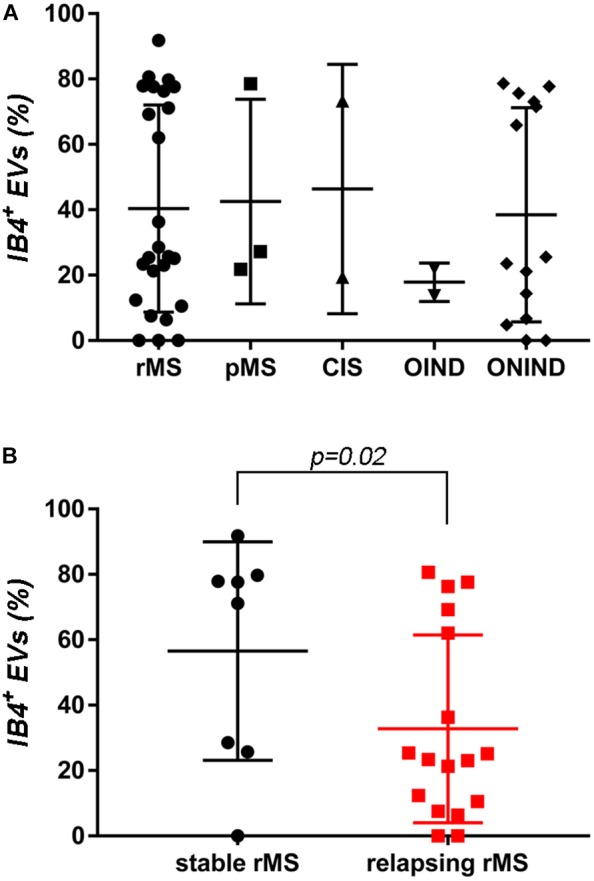
No differences in IB4^+^ CSF-EVs were observed among the examined samples. **(A)** Flow cytometry analysis of IB4^+^ CSF-EVs collected from patients with rMS (*n* = 25), patients with pMS (*n* = 3), patients with pMS (*n* = 2), patients with OIND (*n* = 2), and patients with ONIND (*n* = 14). The Kruskal–Wallis statistical test was used to calculate the reported *p*-values (*p* = 0.83). Individual dots indicate values for single donors. **(B)** Quantitative flow cytometry analysis of CSF-EVs collected from patients with rMS in the clinically stable (*n* = 8) or acute (*n* = 17) phase of the disease. The Mann–Whitney *U*-test was used to calculate the reported *p*-values (*p* = 0.02). Individual dots indicate the values for single donors.

A flow cytometry analysis was conducted on CSF-isolated EVs to determine the presence of IB4, a marker of myeloid origin. Table 3 summarizes demographic and clinical features of our sample. As experimental control we treated with Triton X-100 CSF-EV samples after its IB4 labeling. A reduction in MFI was observed, confirming that our particles are actually lipid structures and that IB4 has its target on membrane of isolated CSF-EVs (Supplementary Figure S2). Figure 2A shows the percentages ± SD of IB4^+^ EVs in the rMS, pMS, CIS, OIND, and ONIND groups. No statistically significant differences emerged from the comparison among the studied groups (*p* = 0.83), similar to the comparison among the other selected clinical and instrumental parameters in the rMS subgroups (data not shown). In contrast, after stratifying subjects with rMS according to clinical relapse, we observed a significant increase in the number of myeloid CSF-EVs in patients with stable rMS (Figure 2B).

**Table 3:** Demographic and clinical features of the population used to determine the fluorescence intensity of IB4^+^ CSF-EVs (EVs/ml).

	Patients		Controls		
		
	rMS	pMS	CIS	OIND°°	ONIND°°°
No. of patients	25	3	2	2	14
Sex (female)	17	0	1	2	9
Age°	34; 23–49	38; 38–44	35; 29–41	59; 52–66	50; 24–84
EDSS°	2.0; 0.0–5.5	1.5; 1.0–6.0	1.5; 1.0–2.0	NA	NA
Disease duration (months)°	14; 1–232	23; 12–114	1; 1–1	NR	NR
No. of subjects in relapse	17	0	1	NA	NA
No. of subjects taking steroids	14	0	1	0	0
No. of subjects with GAD^+^ lesions on MRI	16	0	0	NA	NA


*CSF-EVs, cerebrospinal fluid extracellular vesicles; CIS, clinically isolated syndrome; rMS, relapsing multiple sclerosis; pMS, progressive multiple sclerosis; OIND, other inflammatory neurological disorder; ONIND, other non-inflammatory neurological disorder; EDSS, expanded disability status scale; GAD, gadolinium; MRI, magnetic resonance imaging; NR, not reported; NA, not applicable. °The values are presented as medians; minima-maxima. °°The two subjects with OIND were affected by myelitis. °°°Four of the subjects with ONIND were diagnosed with normal pressure hydrocephalus, four with chronic cerebrovascular disorders, and one each with acute ischaemic stroke, spastic tetraparesis, benign intracranial hypertension, parkinsonism, diabetic polyneuropathy, and conversion disorder.*

### Differences in the Expression of Surface Markers on CSF-EVs in Patients With rMS During the Course of Clinical Relapse and in the Presence of Lesions on Gadolinium-Enhanced MRI

Many studies have reported surface markers that identify Th1 and Th2 cells, as well as their relationship to MS and its mouse model, experimental autoimmune encephalomyelitis (EAE). For this reason, we determined the concentration of EVs positive for several CDs in the CSF of 25 patients with MS, 2 patients with CIS, and 13 controls using FACS analyzes; the demographic and clinical features are summarized in Table 4. Table 1 summarizes CDs selected for our analysis. Briefly, Th1 and Th2 subsets of CD4^+^ lymphocytes are characterized by the expression of some chemokine receptors. In particular, the chemokine receptor CCR5 is associated with the Th1 phenotype, whereas CCR3 is expressed preferentially on activated Th2 cells (Bonecchi et al., 1998; Scotet et al., 2001).

**Table 4:** Demographic and clinical features of the population used to determine the percentages of CSF-EVs positive for the CDs investigated.

	Patients		Controls		
		
	rMS	pMS	CIS	OIND°°	ONIND°°°
No. of patients	23	2	2	1	12
Sex (female)	14	0	1	1	9
Age°	35, 17, 62	41, 38, 44	35; 29–41	66	50, 24, 84
EDSS°	2.0-0.0-4.5	3.75-1.5-6.0	1.5; 1.0–2.0	NA	NA
Disease duration (months)°	17, 1, 360	17.5, 12, 23	1; 1–1	NR	NR
No. of subjects in relapse	18	0	1	NA	NA
No. of subjects taking steroids	15	0	1	0	0
No. of subjects with GAD^+^ lesions on MRI	13	0	0	NA	NA


*CSF-EVs, cerebrospinal fluid extracellular vesicles; CDs, clusters of differentiation; CIS, clinically isolated syndrome; rMS, relapsing multiple sclerosis; pMS, progressive multiple sclerosis; OIND, other inflammatory neurological disorder; ONIND, other non-inflammatory neurological disorder; EDSS, expanded disability status scale; GAD, gadolinium; MRI, magnetic resonance imaging; NR, not reported; NA, not applicable. °The values are presented as medians; minima-maxima. °°The subject with OIND was affected by myelitis. °°°Three of the subjects with ONIND were diagnosed with chronic cerebrovascular disorders, two with normal pressure hydrocephalus, and one each with acute ischaemic stroke, benign intracranial hypertension, parkinsonism, spastic paraparesis, diabetic polyneuropathy, brain tumor outcomes, and conversion disorder.*

**FIGURE 3:**
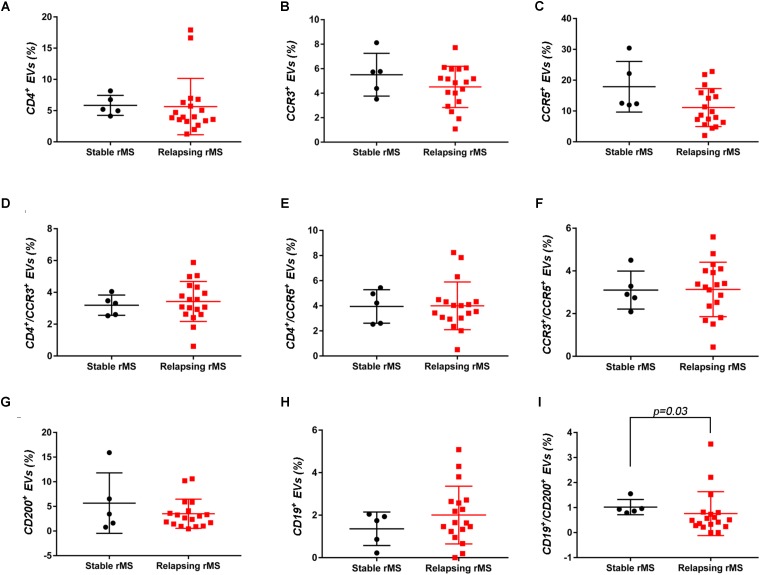
Differences in extracellular vesicles between patients with rMS in the course of a clinical relapse and stable phase. Flow cytometry analysis of extracellular vesicles stained for the selected CDs in CSF collected from patients with rMS in the clinically stable (*n* = 5) or acute (*n* = 18) phase of the disease. The Mann–Whitney *U*-test was used to calculate the reported *p*-values: **(A)** CD4 (*p* = 0.20), **(B)** CCR3 (*p* = 0.40), **(C)** CCR5 (*p* = 0.09), **(D)** CD4/CCR3 (*p* = 0.59), **(E)** CD4/CCR5 (*p* = 0.86), **(F)** CCR3/CCR5 (*p* = 0.86), **(G)** CD200 (*p* = 0.64), **(H)** CD19 (*p* = 0.48), and **(I)** CD19/CD200 (*p* = 0.03). Individual dots indicate the values for single donors.

**FIGURE 4:**
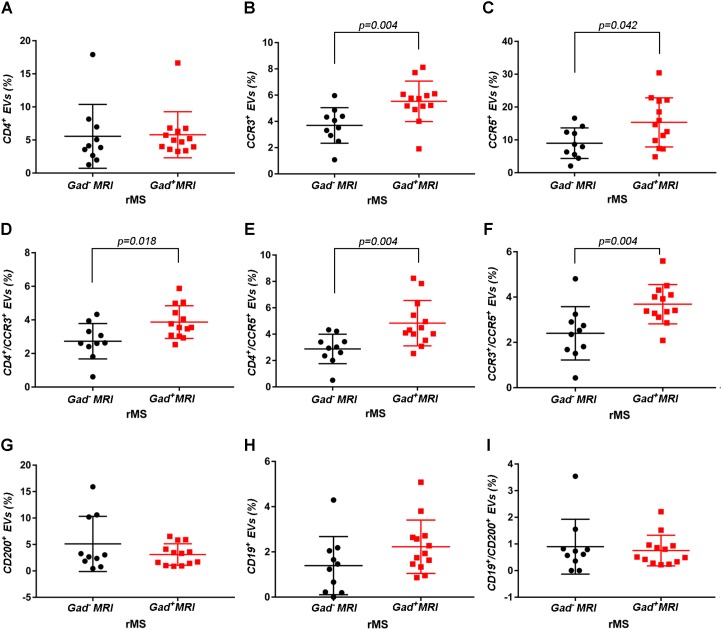
Differences in extracellular vesicles between patients with rMS in the course of a neuroradiological relapse and the stable phase. Flow cytometry analysis of extracellular vesicles stained for the selected CDs in CSF collected from patients with rMS in the neuroradiologically stable (*n* = 10) or active (*n* = 13) phase of the disease. The Mann–Whitney *U*-test was used to calculate the reported *p*-values: **(A)** CD4 (*p* = 0.52), **(B)** CCR3 (*p* = 0.004), **(C)** CCR5 (*p* = 0.042), **(D)** CD4/CCR3 (*p* = 0.018), **(E)** CD4/CCR5 (*p* = 0.004), **(F)** CCR3/CCR5 (*p* = 0.004), **(G)** CD200 (*p* = 0.83), **(H)** CD19 (*p* = 0.08), **(I)** CD19/CD200 (*p* > 1). Individual dots indicate the values for single donors.

**FIGURE 5:**
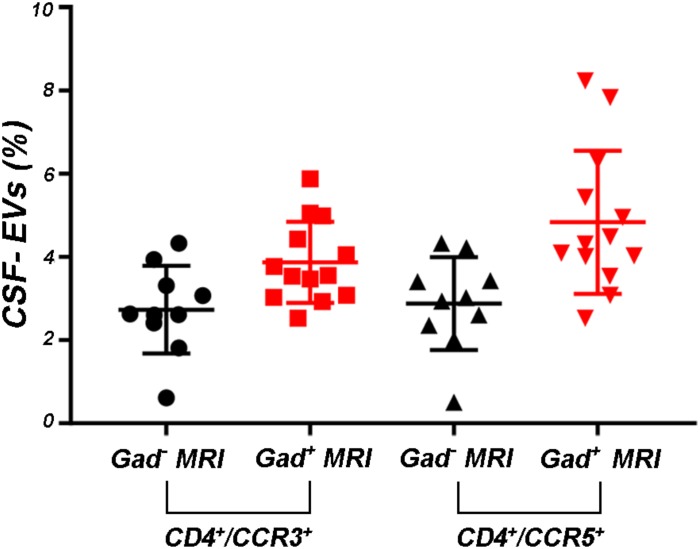
Levels of CD4^+^/CCR5^+^ CSF-EVs were increased in patients with rMS presenting with gadolinium-positive lesions. Flow cytometry analysis of CD4^+^/CCR3^+^ and CD4^+^/CCR5^+^ CSF-EVs in patients with rMS in the neuroradiologically stable (*n* = 10) or active (*n* = 13) phase of the disease. The Mann–Whitney *U*-test was used to calculate the reported *p*-values between gadolinium-positive patients with rMS (*p* = 0.13). Individual dots indicate the values for single donors.

Supplementary Figures S3A–F shows the mean percentages ± SD of CD4^+^, CCR3^+^, CCR5^+^, CD4^+^/CCR3^+^, CD4^+^/CCR5^+^, and CCR3^+^/CCR5^+^ CSF-EVs in all examined groups. We did not observe statistically significant differences in the investigated markers among the five studied groups. Furthermore, after pooling the rMS subgroup according to some of the selected clinical and instrumental parameters (i.e., steroid intake and disease duration), similar concentrations of all markers were observed (data not shown). Additionally, after stratifying subjects with rMS according to clinical relapse, we did not obtain any statistically significant differences in the levels of these markers (Figures 3A–F). In contrast, in the presence of brain and spine lesions on gadolinium-enhanced MRI, regardless of the presence of clinical symptoms and signs of a relapse, subjects with rMS showed a statistically significant increase in the numbers of CCR3^+^ (*p* = 0.004), of CCR5^+^ (*p* = 0.042), CD4^+^/CCR3^+^ (*p* = 0.018), CD4^+^/CCR5^+^ (*p* = 0.004), and CC3^+^/CCR5^+^ (*p* = 0.004) CSF-EVs (Figures 4A–F). We also observed an increased number of CD4^+^/CCR5^+^ (Th1) CSF-EVs compared to CD4^+^/CCR3^+^ (Th2) CSF-EVs in patients with gadolinium-positive lesions (Figure 5).

Two other markers examined were CD19, one of the main membrane proteins of B lymphocytes, which is expressed in the maturation step (Stüve et al., 2014), and CD200, which is expressed on neurons, oligodendrocytes and on reactive astrocytes (Koning et al., 2009). Supplementary Figures S3G–I shows the mean percentages ± SD of CD200^+^, CD19^+^, and CD19^+^/CD200^+^ CSF-EVs in the rMS, pMS, CIS, OIND, and ONIND groups. We did not observe statistically significant differences in the comparisons among the five studied groups. Similarly, an evaluation of the markers in the rMS subgroups stratified according to steroid intake or disease duration showed similar concentrations (data not shown). Instead, we observed a statistically significant reduction in the number of CD19^+^/CD200^+^ CSF-EVs in subjects with in the course of a clinical relapse (*p* = 0.030) (Figure 3I), whereas we observed differences in these two markers between subjects with rMS stratified according to the presence of gadolinium-enhanced lesions, but these differences did not reach statistical significance (Figures 4G–I).

## Discussion

The aims of this work were to confirm the presence of circulating EVs in CSF, to identify some of the cells from which they originated, and to investigate the possible use of these CSF-EVs as biomarkers for both the diagnosis and determination of the active status of MS.

First, consistent with the literature, our data confirm the presence of EVs in the CSF (Supplementary Figure S1). Moreover, the EV concentration is not a useful parameter for differentiating MS from ONDs (Figure 1A). This result is not surprising, as EVs are released by both inflammatory and non-inflammatory cells, which might be involved in different pathological conditions. A non-significant increase in the EV concentration was observed in patients with pMS and patients with CIS compared to patients with rMS (Figure 1A). In contrast, a statistically significant difference in the EV content was observed in patients with rMS stratified according to the two different clinical MS phases (i.e., stable or relapsing), with a higher concentration observed in relapsing subjects (Figure 1B). These results are consistent with findings reported by Minagar et al. (2001) and Verderio et al. (2012).

In the absence of specific cell-origin CSF-EV markers for MS, we decided to investigate the presence of markers derived from different cell types that could be involved in MS pathology (e.g., lymphocytes and myeloid cells) on EVs. One of the first examined markers was IB4, an isolectin that labels microglia (Figure 2A). Our results appear to be somewhat inconsistent with the findings reported by Verderio et al. (2012). Indeed, we observed a higher concentration of IB4^+^ CSF-EVs in subjects with rMS who were in the stable phase compared subjects in the relapsing phase (Figure 2B). The differences observed between these two studies could be correlated to the lower size of our population, or to different settings of FACS.

Although the precise etiology of MS has not yet been thoroughly elucidated, many researchers postulate that it is an autoimmune disease characterized by the infiltration, accumulation and activation of myelin-specific T lymphocytes and macrophages in the CNS. In this inflammatory cascade, CD4^+^ T lymphocytes play a central role in the pathogenesis of MS, predominating in acute lesions (Chitnis, 2007), and chemokine receptors participate in their recruitment into the CNS. For this reason, we evaluated the presence of Th cell markers (i.e., CD4, CCR3, and CCR5) on circulating CSF-EVs. CCR3 and CCR5 are also expressed on foamy macrophages and activated microglia in chronic active MS plaques, as well as on astrocytes, particularly astrocytes forming processes around vessels and at the glia limitans. The two chemokine receptors, particularly CCR5, are also present on large numbers of infiltrating lymphocytes (Simpson et al., 2000; Trebst et al., 2001). Moreover, in C57BL6 mice, CCR5 knockout suppresses EAE (Gu et al., 2016). In our cohort of patients with rMS, a significant increase in CD4^+^/CCR5^+^ CSF-EVs was observed in the presence of gadolinium-positive lesions compared to gadolinium-negative lesions (Figure 4E). This result was consistent with previous papers showing that Th1 lymphocytes are enriched in patients with MS and exhibit proinflammatory activity (Balashov et al., 1999; Zang et al., 2000). Furthermore, several studies have reported Th1 dominance over Th2 cells among peripheral blood lymphocytes and in the CSF of patients in the active phase of MS and in active demyelinating MS brain lesions (Balashov et al., 1999; Sørensen et al., 1999; Uzawa et al., 2010). Consistent with these data, we also observed an increased number of CD4^+^/CCR5^+^ CSF-EVs compared to CD4^+^/CCR3^+^ CSF-EVs in radiologically relapsing patients, although the increase was not statistically significant (Figure 5).

For decades, MS was thought to be a CD4^+^ T-cell mediated disease. However, interest has recently developed in the involvement of CD8^+^ T cells. Indeed, a large number of CD8^+^ T cells are present in CNS lesions (Babbe et al., 2000), and these cells appear to have a mixed functional phenotype (i.e., expression of cytotoxic/inflammatory, regulatory, and effector molecules) (Crawford et al., 2004; Baughman et al., 2011; Cunnusamy et al., 2014). In the present study, we observed a significantly higher level of CCR3^+^/CCR5^+^ EVs (Figure 4F). The coexpression of these two markers on the cell surface has been observed in a new subset of activated CD8 T cells (Ferguson and Engelhard, 2010), but the involvement of these cells in MS immunopathology has never been described.

In patients with MS, B cells are believed to cross the blood–brain barrier and undergo stimulation, antigen-driven affinity maturation and clonal expansion within the supportive CNS environment (Hauser, 2015), playing a major role in mediating tissue damage (Kuenz et al., 2008). The role of B cells is primarily to reduce MS activity induced by the administration of an anti-B lymphocyte monoclonal antibody (Baker et al., 2017). In our study, consistent with B cell involvement in MS, we observed a non-significant increase in the number of CD19^+^ EVs in both clinically and radiologically relapsing patients (Figures 3H, 4H).

Glial activation in patients with MS might be due to alterations in neuronal–glial and/or glial–glial crosstalk, and these alterations involve the CD200-CD200R system. The involvement of this pathway in MS pathology was reported by Valente et al. In fact, the authors showed that EAE is more severe in CD200^-/-^ mice than in wild type mice (Valente et al., 2017). In contrast, the attenuated disease observed in EAE Wld^s^ mice was associated with robust constitutive expression of the CD200 molecule on neurons in the CNS compared with control mice (Chitnis et al., 2007). A reduction in the CD200 level was also observed on astrocytes in the center and rim of MS lesions (Koning et al., 2007), suggesting that CD200 might be involved in disease progression. Moreover, CD200R is restricted to cells of the myeloid lineage (Hoek et al., 2000; Wright et al., 2000; Barclay et al., 2002) and is responsible for changing the phenotype of these cells from M1-like to M2-like (Lue et al., 2010). For this reason, CD200 appears to be an interesting marker. To the best of our knowledge, we are the first group to determine CD200 levels on the EV surface and show a decrease in clinically and radiologically relapsing subjects, even if the decrease was not statistically significant (Figures 3G, 4G). Due to their predicted involvement in glial activation and in the onset of clinical MS symptoms, CD200^+^ CSF-EVs must be examined in further investigations.

We have also observed significantly lower levels of CD19^+^/CD200^+^ CSF-EVs in our patients with MS during a clinically active phase than in patients in the stable phase (Figure 3I). CD19 and CD200 are both expressed on immature and naïve B lymphocytes (Rasmussen et al., 2015). This double staining has never been associated with MS, but a reduction in the number of CD19/CD200 B lymphocytes has recently been described in the acute phase of severe alopecia areata, a hair follicle-specific Th1 cell-mediated autoimmune disease (Gilhar and Kalish, 2006; Ma et al., 2017).

**FIGURE 6:**
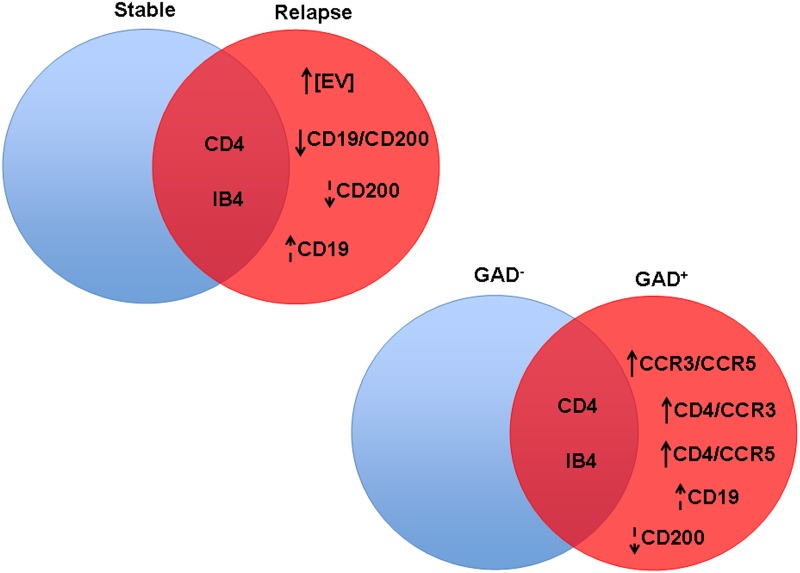
Significant differences in CSF-EV biomarkers among patients with rMS presenting different clinical and radiological statuses. Findings are summarized. The indicated biomarkers were different between comparison groups. The arrow preceding the name of each biomarker indicates an increase or decrease in the CSF-EV concentration in rMS groups, whereas the lack of an arrow indicates an invariant value. Solid arrows indicate statistically significant differences, and dotted arrows indicate non-significant differences.

All of the discussed results are summarized in Figure 6.

The present study provides evidence that the CSF-EV concentration is not a useful diagnostic parameter to differentiate MS from other neurological diseases. In contrast, certain markers selected to identify the cellular origin of CSF-EVs were differentially expressed during the relapsing phase of MS compared to the stable state of MS in the investigated rMS cohort. These differences were observed both during clinical relapse and in the presence of brain or spine lesions on gadolinium-enhanced MRI, suggesting that only some of the biological processes occurring during both plaque formation and the appearance of clinical symptoms are the same. This apparent “clinico-radiological paradox” has been recently observed in a study showing that platelet-derived growth factor levels in the CSF predict a prolonged relapse-free period in patients with MS but not the lack of lesions on gadolinium-enhanced MRI (Stampanoni Bassi et al., 2018).

The main weakness of our study could be the low number of included subjects, even if this number is similar to other studies of MS, and although our data must be confirmed in different and larger MS cohorts, CSF-EV observations might constitute a crucial prognostic tool that would be of benefit to further targeted projects and to the selection of the most appropriate therapy.

## Ethics Statement

This study was approved by the Ethics Committee of the Medical School of the University of Palermo (Palermo 1). All subjects provided written informed consent in accordance with the Declaration of Helsinki.

## Author Contributions

FG, PR, GSc, GSav, and GSal conceived and designed the experiments. FG, MB, and EA performed flow cytometry analyzes. PR, SR, MM, GV, GSav, and GSal selected patients, performed lumbar puncture and collected and stored cerebrospinal fluid samples. PR, GSav, and GSal analyzed the data. FG, PR, and GSal wrote the manuscript. All authors have read and approved the final version of the manuscript.

## Conflict of Interest Statement

The authors declare that the research was conducted in the absence of any commercial or financial relationships that could be construed as a potential conflict of interest.

## Footnotes

1 https://figshare.com/s/149f680f08d0d512c708

## Supplementary Material

FIGURE S1 Characterization of EVs isolated from CSF. **(A)** Flow cytometry analysis of isolated CSF-EVs. SSC, side scatter; FSC, forward scatter. For the size determination, polystyrene microspheres were used. **(B)** Representative graphs showing the size distributions and concentrations of CSF-EV as measured by nanoparticle tracking analysis (NTA) and **(C)** particles moving under Brownian motion. **(D)** Representative Transmission electron microscopic image of CSF-EVs. Scale bar represents 200 nm. **(E)** Flow cytometry histogram of CSF-EVs after anti-Hsp70-FITC conjugated antibodies staining. **(F)** Acetylcholinesterase activity assay. **(G)** Flow cytometry histogram of CSF-EVs after Annexin-V staining. **(H)** Pyronin Y labelling of CSF-EVs.

FIGURE S2 CSF-EVs are detergent soluble. CSF-EVs stained with FITC-IB4 measured before (blue) and after (green) treatment with 0.1% Triton X-100, showing that the detected events are detergent-soluble.

FIGURE S3 Differences in extracellular vesicles between patients with multiple sclerosis and patients with other neurological disorders. Flow cytometry analysis of extracellular vesicles stained for the selected CDs in CSF collected from patients with rMS (*n* = 23), patients with pMS (*n* = 2), patients with CIS (*n* = 2), patients with OIND (*n* = 1) and patients with ONIND (*n* = 12). The Kruskal–Wallis statistical test was used to calculate the reported *p*-values: **(A)** CD4 (*p* = 0.74), **(B)** CCR3 (*p* = 0.12), **(C)** CCR5 (*p* = 0.18), **(D)** CD4/CCR3 (*p* = 0.25), **(E)** CD4/CCR5 (*p* = 0.25), **(F)** CCR3/CCR5 (*p* = 0.93), **(G)** CD200 (*p* = 0.25), **(H)** CD19 (*p* = 0.25), and **(I)** CD19/CD200 (*p* = 0.25). Individual dots indicate the values for single donors.

TABLE S1 Demographic and clinical features of patients with rMS at the moment of the lumbar puncture.
