# Supplementary material for: Differences in Intercellular Communication During Clinical Relapse and Gadolinium-Enhanced MRI in Patients With Relapsing Remitting Multiple Sclerosis: A Study of the Composition of Extracellular Vesicles in Cerebrospinal Fluid
Source: Front Cell Neurosci. 2018 Nov 15;12:418. doi: 10.3389/fncel.2018.00418 (PMC6249419; doi:10.3389/fncel.2018.00418)
Supplement: FIGURE S1 — Characterization of EVs isolated from CSF. (A) Flow cytometry analysis of isolated CSF-EVs. SSC, side scatter; FSC, forward scatter. For the size determination, polystyrene microspheres were used. (B) Representative graphs showing the size distributions and concentrations of CSF-EV as measured by nanoparticle tracking analysis (NTA) and (C) particles moving under Brownian motion. (D) Representative Transmission electron microscopic image of CSF-EVs. Scale bar represents 200 nm. (E) Flow cytometry histogram of CSF-EVs after anti-Hsp70-FITC conjugated antibodies staining. (F) Acetylcholinesterase activity assay. (G) Flow cytometry histogram of CSF-EVs after Annexin-V staining. (H) Pyronin Y labelling of CSF-EVs. [file Image_1.pdf]

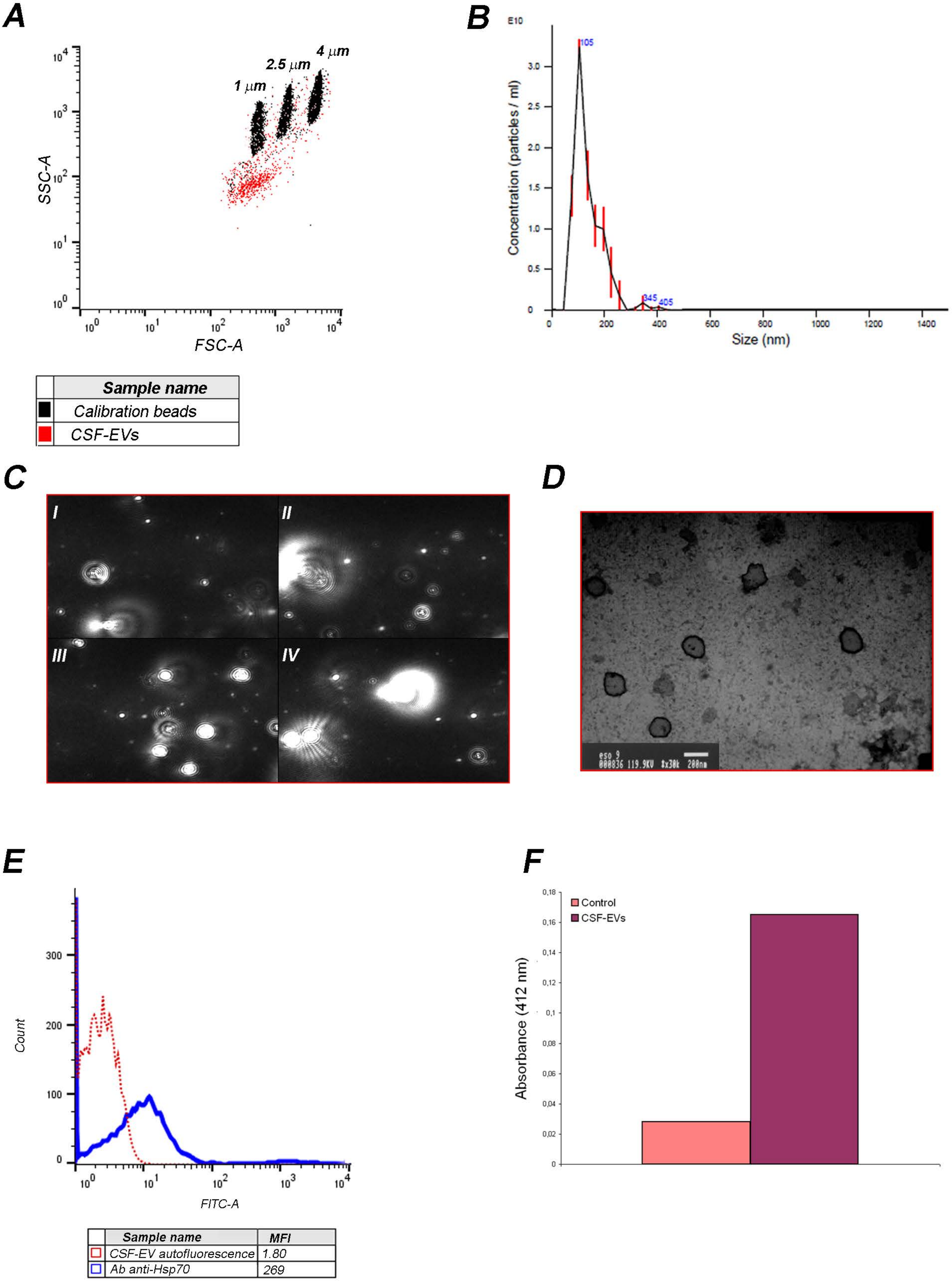

A
SSC-A
FSC-A
1 µm
2.5 µm
4 µm
Sample name
Calibration beads
CSF-EVs
B
E10
Concentration (particles / ml)
Size (nm)
105
345
405
C
I
II
III
IV
D
eso 9
000836 119.9KV $x30k 200nm
E
Count
FITC-A
Sample name
MFI
CSF-EV autofluorescence
1.80
Ab anti-Hsp70
269
F
Control
CSF-EVs
Absorbance (412 nm)

G

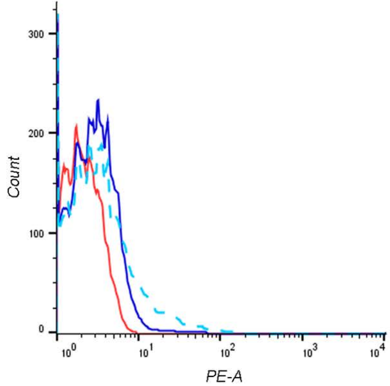

	Sample name	MFI	% Annexin V[+] EVs
□	CSF-EV autofluorescence	1.53	0.07
□	Annexin V w Ca^{2+}	3.35	6.91
□	Annexin V w/o Ca^{2+}	1.97	0.93

H

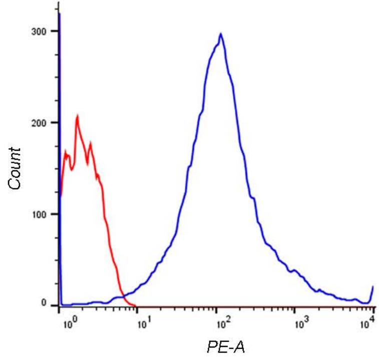

	Sample name	MFI
□	CSF-EV autofluorescence	1.53
□	Pyronin Y	264
