# Supplementary figures and images for: Differences in Intercellular Communication During Clinical Relapse and Gadolinium-Enhanced MRI in Patients With Relapsing Remitting Multiple Sclerosis: A Study of the Composition of Extracellular Vesicles in Cerebrospinal Fluid
Source: Front Cell Neurosci. 2018 Nov 15;12:418. doi: 10.3389/fncel.2018.00418 (PMC6249419; doi:10.3389/fncel.2018.00418)

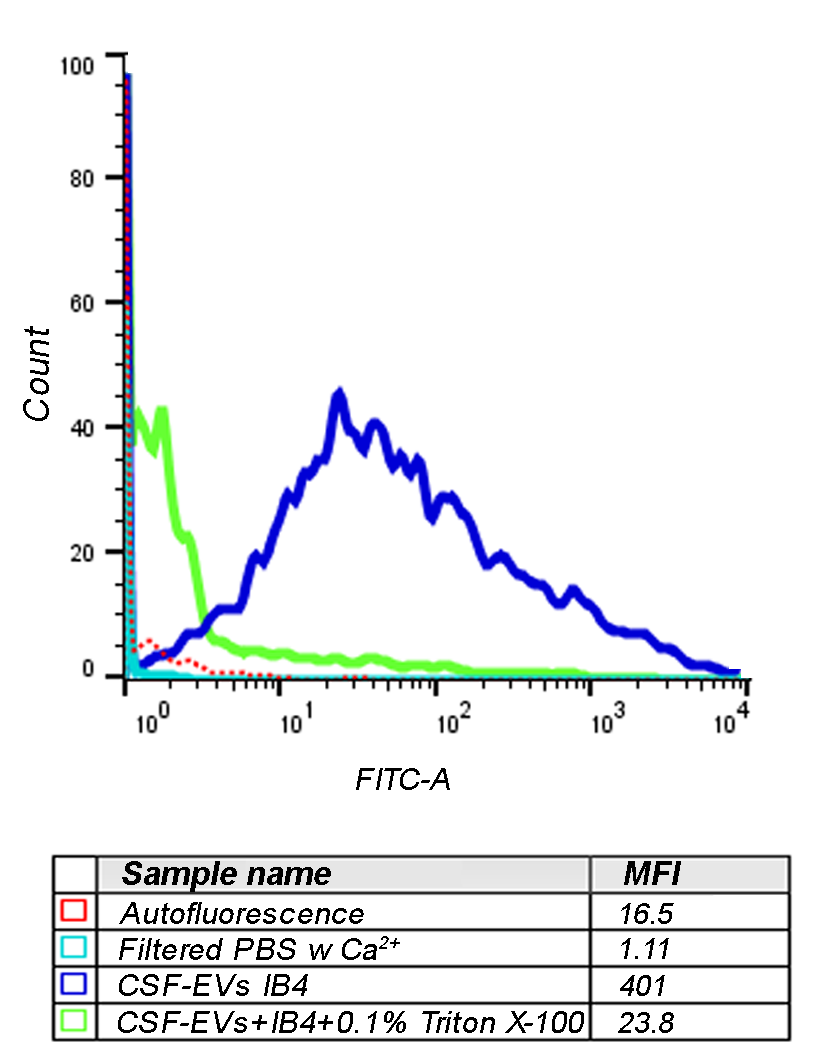

Supplement: FIGURE S2 — CSF-EVs are detergent soluble. CSF-EVs stained with FITC-IB4 measured before (blue) and after (green) treatment with 0.1% Triton X-100, showing that the detected events are detergent-soluble. [file Image_2.tif]

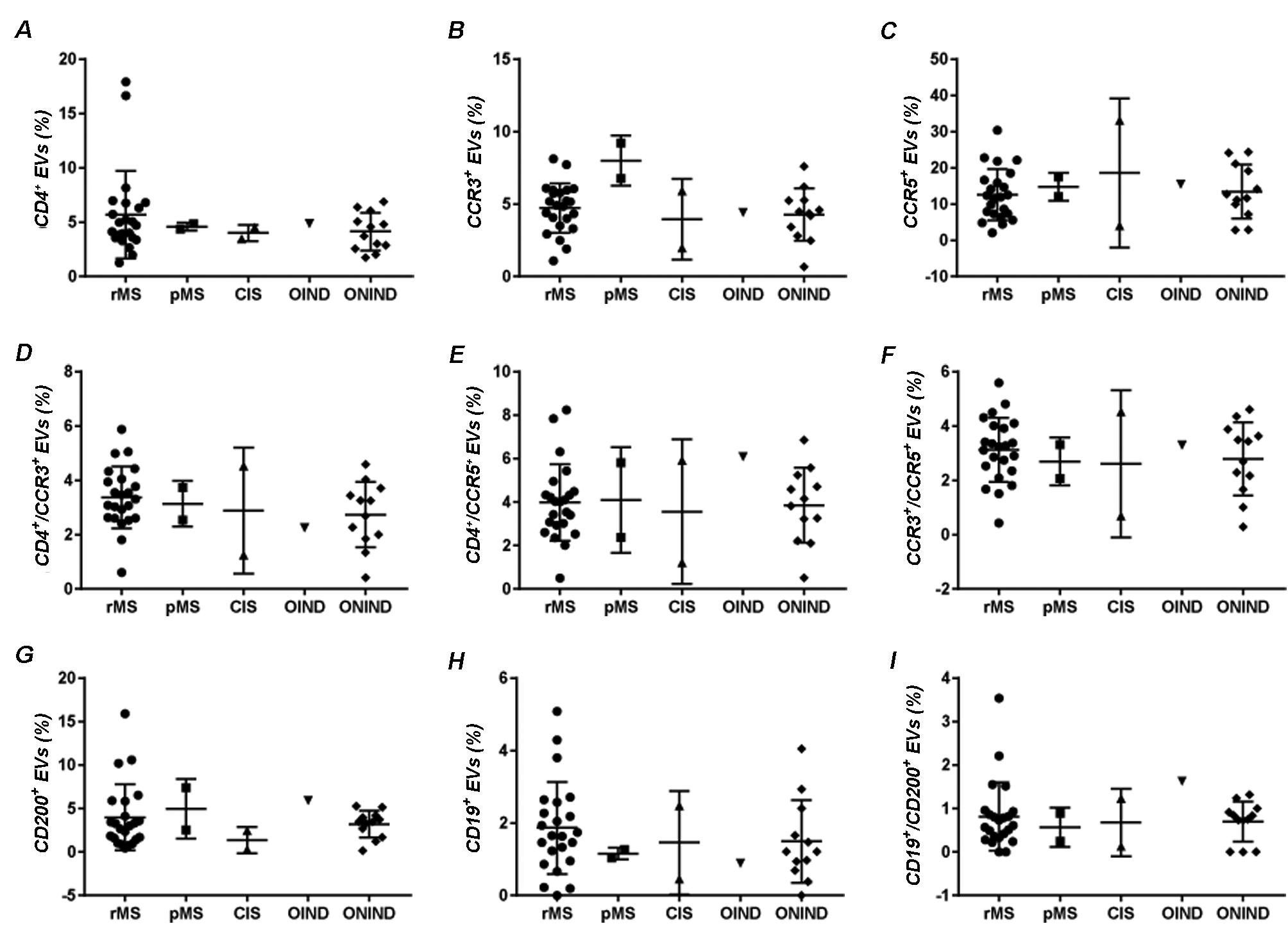

Supplement: FIGURE S3 — Differences in extracellular vesicles between patients with multiple sclerosis and patients with other neurological disorders. Flow cytometry analysis of extracellular vesicles stained for the selected CDs in CSF collected from patients with rMS (n = 23), patients with pMS (n = 2), patients with CIS (n = 2), patients with OIND (n = 1) and patients with ONIND (n = 12). The Kruskal–Wallis statistical test was used to calculate the reported p-values: (A) CD4 (p = 0.74), (B) CCR3 (p = 0.12), (C) CCR5 (p = 0.18), (D) CD4/CCR3 (p = 0.25), (E) CD4/CCR5 (p = 0.25), (F) CCR3/CCR5 (p = 0.93), (G) CD200 (p = 0.25), (H) CD19 (p = 0.25), and (I) CD19/CD200 (p = 0.25). Individual dots indicate the values for single donors. [file Image_3.tif]
