# Supplementary material for: Differences in Intercellular Communication During Clinical Relapse and Gadolinium-Enhanced MRI in Patients With Relapsing Remitting Multiple Sclerosis: A Study of the Composition of Extracellular Vesicles in Cerebrospinal Fluid
Source: Front Cell Neurosci. 2018 Nov 15;12:418. doi: 10.3389/fncel.2018.00418 (PMC6249419; doi:10.3389/fncel.2018.00418)
Supplement: TABLE S1 — Demographic and clinical features of patients with rMS at the moment of the lumbar puncture. [file Table_1.DOC]

**Supplemental Table 1**. Demographic and clinical features of patients with rMS at the moment of the lumbar puncture.

| Subject No. | Sex | Date of LP | Age | MS duration (months) | EDSS at LP | Status | Date of MRI | GAD status at MRI | Steroid intake |
| --- | --- | --- | --- | --- | --- | --- | --- | --- | --- |
| 1 | F | 27/01/2015 | 34 | 89 | 0.0 | Relapsing | 13/01/2015 | Gad^-^ | Y |
| 2 | F | 11/09/2013 | 49 | 1 | 3.5 | Relapsing | 04/09/2013 | Gad^+^ | Y |
| 3 | M | 24/03/2014 | 39 | 9 | 4.0 | Relapsing | 11/03/2014 | Gad^+^ | Y |
| 4 | F | 04/12/2012 | 48 | 29 | 4.5 | Relapsing | 22/11/2012 | Gad^+^ | Y |
| 5 | F | 09/12/2014 | 35 | 47 | 2.0 | Relapsing | 25/11/2014 | Gad^+^ | Y |
| 6 | F | 04/06/2015 | 39 | 4 | 2.0 | Stable | 21/05/2015 | Gad^-^ | N |
| 7 | F | 16/07/2012 | 27 | 122 | 2.5 | Stable | 02/07/2012 | Gad^+^ | N |
| 8 | F | 24/03/2014 | 47 | 1 | 3.0 | Relapsing | 20/03/2014 | Gad^-^ | Y |
| 9 | F | 29/05/2013 | 30 | 5 | 0.0 | Stable | 28/05/2013 | Gad^-^ | N |
| 10 | F | 10/06/2014 | 17 | 25 | 2.0 | Relapsing | 27/05/2014 | Gad^-^ | Y |
| 11 | M | 05/02/2015 | 23 | 17 | 2.0 | Relapsing | 22/01/2015 | Gad^-^ | Y |
| 12 | F | 17/12/2012 | 34 | 1 | 3.5 | Relapsing | 11/12/2012 | Gad^+^ | Y |
| 13 | F | 05/03/2012 | 30 | 3 | 5.5 | Relapsing | 21/02/2012 | Gad^+^ | Y |
| 14 | M | 12/06/2014 | 37 | 1 | 2.0 | Relapsing | 05/06/2014 | Gad^+^ | Y |
| 15 | F | 13/05/2014 | 39 | 3 | 3.5 | Relapsing | 29/04/2014 | Gad^+^ | N |
| 16 | M | 22/05/2014 | 32 | 115 | 1.0 | Stable | 13/05/2014 | Gad^-^ | N |
| 17 | F | 18/11/2014 | 18 | 4 | 0.0 | Stable | 04/11/2014 | Gad^-^ | N |
| 18 | F | 21/05/2012 | 35 | 12 | 1.0 | Relapsing | 20/05/2012 | Gad^-^ | Y |
| 19 | F | 10/02/2015 | 33 | 232 | 1.0 | Stable | 28/01/2015 | Gad^+^ | N |
| 20 | M | 12/06/2014 | 28 | 37 | 2.0 | Relapsing | 29/05/2014 | Gad^-^ | N |
| 21 | F | 04/04/2012 | 32 | 2 | 3.5 | Relapsing | 02/04/2012 | Gad^+^ | N |
| 22 | F | 12/07/2012 | 45 | 14 | 2.0 | Stable | 10/07/2012 | Gad^-^ | N |
| 23 | M | 04/12/2014 | 34 | 10 | 2.5 | Relapsing | 20/11/2014 | Gad^+^ | Y |
| 24 | M | 26/09/2014 | 36 | 3 | 4.0 | Stable | 12/09/2014 | Gad^-^ | N |
| 25 | F | 04/12/2012 | 39 | 115 | 2.0 | Relapsing | 29/11/2012 | Gad^-^ | Y |
| 26 | F | 17/01/2013 | 18 | 6 | 1.0 | Relapsing | 08/01/2013 | Gad^+^ | Y |
| 27 | F | 10/06/2014 | 49 | 2 | 3.0 | Relapsing | 27/05/2014 | Gad^+^ | N |
| 28 | F | 21/01/2013 | 33 | 11 | 2.0 | Stable | 07/01/2013 | Gad^-^ | N |
| 29 | M | 23/09/2015 | 39 | 178 | 0.0 | Stable | 10/09/2015 | Gad^+^ | N |
| 30 | M | 22/01/2013 | 35 | 27 | 3.5 | Stable | 08/01/2013 | Gad^+^ | N |
| 31 | M | 15/06/2012 | 26 | 3 | 3.5 | Stable | 01/06/2012 | Gad^+^ | N |
| 32 | F | 24/02/2012 | 29 | 1 | 1.0 | Relapsing | 20/02/2012 | Gad^+^ | Y |
| 33 | F | 16/03/2012 | 62 | 360 | 3.5 | Relapsing | 15/03/2012 | Gad^+^ | Y |
| 34 | F | 09/09/2013 | 24 | 1 | 1.0 | Relapsing | 06/09/2013 | Gad^-^ | Y |
| 35 | F | 12/05/2014 | 29 | 46 | 1.5 | Relapsing | 29/04/2014 | Gad^+^ | Y |
|  |  |  |  |  |  |  |  |  |  |

Abbreviations: rMS relapsing multiple sclerosis, LP lumbar puncture, M male, F female, EDSS expanded disability status scale, MRI magnetic resonance imaging, Gad gadolinium diethylenetriamine penta-acetic acid, Gad^+^ presence of T1 lesions in the brain or spinal cord on gadolinium-enhanced MRI, Gad^-^ absence of T1 lesions in the brain or spinal cord on gadolinium-enhanced MRI, Y yes, N no.
